# Supplementary material for: Modeling glioblastoma heterogeneity as a dynamic network of cell states
Source: Mol Syst Biol. 2021 Sep 16;17(9):e10105. doi: 10.15252/msb.202010105 (PMC8444284; doi:10.15252/msb.202010105)
Supplement: Supplementary file 6 — Source Data for Figure 5 [file MSB-17-e10105-s004.zip › Figure5A_sourcedata/GSEA_3071/hallmarks_stateV.GseaPreranked.1623742546329/HALLMARK_UV_RESPONSE_DN.html]

Details for gene set HALLMARK\_UV\_RESPONSE\_DN[GSEA]

|  || Dataset | state43071 |
| Phenotype | NoPhenotypeAvailable |
| Upregulated in class | na\_neg |
| GeneSet | HALLMARK\_UV\_RESPONSE\_DN |
| Enrichment Score (ES) | -0.52977985 |
| Normalized Enrichment Score (NES) | -2.8610196 |
| Nominal p-value | 0.0 |
| FDR q-value | 0.0 |
| FWER p-Value | 0.0 |
Table: GSEA Results Summary

  

Fig 1: Enrichment plot: HALLMARK\_UV\_RESPONSE\_DN      
 Profile of the Running ES Score & Positions of GeneSet Members on the Rank Ordered List

  

| PROBE | GENE SYMBOL | GENE\_TITLE | RANK IN GENE LIST | RANK METRIC SCORE | RUNNING ES | CORE ENRICHMENT || 1 | CELF2 |  |  | 137 | 1.300 | -0.0087 | No |
| 2 | AMPH |  |  | 164 | 1.238 | 0.0031 | No |
| 3 | MMP16 |  |  | 241 | 1.128 | 0.0038 | No |
| 4 | NFIB |  |  | 306 | 1.062 | 0.0059 | No |
| 5 | INPP4B |  |  | 319 | 1.047 | 0.0178 | No |
| 6 | PLPP3 |  |  | 323 | 1.042 | 0.0314 | No |
| 7 | CDKN1B |  |  | 360 | 0.997 | 0.0380 | No |
| 8 | SRI |  |  | 599 | 0.801 | 0.0032 | No |
| 9 | APBB2 |  |  | 617 | 0.790 | 0.0106 | No |
| 10 | SFMBT1 |  |  | 636 | 0.781 | 0.0177 | No |
| 11 | RBPMS |  |  | 855 | 0.676 | -0.0149 | No |
| 12 | COL1A2 |  |  | 1028 | 0.614 | -0.0396 | No |
| 13 | HAS2 |  |  | 1067 | 0.603 | -0.0387 | No |
| 14 | TGFBR3 |  |  | 1203 | 0.560 | -0.0570 | No |
| 15 | DYRK1A |  |  | 1265 | 0.541 | -0.0614 | No |
| 16 | PIK3R3 |  |  | 1327 | 0.526 | -0.0659 | No |
| 17 | RASA2 |  |  | 1396 | 0.507 | -0.0721 | No |
| 18 | IGF1R |  |  | 1430 | 0.497 | -0.0717 | No |
| 19 | DLG1 |  |  | 1491 | 0.482 | -0.0767 | No |
| 20 | ARHGEF9 |  |  | 1493 | 0.482 | -0.0703 | No |
| 21 | VAV2 |  |  | 1528 | 0.475 | -0.0704 | No |
| 22 | TENT4A |  |  | 2014 | 0.386 | -0.1582 | No |
| 23 | CDON |  |  | 2027 | 0.384 | -0.1553 | No |
| 24 | SCHIP1 |  |  | 2028 | 0.383 | -0.1501 | No |
| 25 | BMPR1A |  |  | 2137 | 0.369 | -0.1658 | No |
| 26 | PRKAR2B |  |  | 2138 | 0.369 | -0.1608 | No |
| 27 | VLDLR |  |  | 2236 | 0.358 | -0.1746 | No |
| 28 | AKT3 |  |  | 2292 | 0.351 | -0.1804 | No |
| 29 | NR1D2 |  |  | 2349 | 0.342 | -0.1865 | No |
| 30 | MGMT |  |  | 2678 | 0.302 | -0.2453 | No |
| 31 | MAP2K5 |  |  | 2718 | 0.298 | -0.2487 | No |
| 32 | PLCB4 |  |  | 2992 | 0.270 | -0.2974 | No |
| 33 | KALRN |  |  | 3306 | -0.268 | -0.3538 | No |
| 34 | ZMIZ1 |  |  | 3488 | -0.297 | -0.3845 | No |
| 35 | ITGB3 |  |  | 3508 | -0.301 | -0.3841 | No |
| 36 | GJA1 |  |  | 3602 | -0.316 | -0.3976 | No |
| 37 | BHLHE40 |  |  | 3620 | -0.318 | -0.3966 | No |
| 38 | SYNJ2 |  |  | 3787 | -0.347 | -0.4237 | No |
| 39 | IRS1 |  |  | 3971 | -0.383 | -0.4536 | No |
| 40 | ATP2B1 |  |  | 4126 | -0.416 | -0.4775 | No |
| 41 | SMAD7 |  |  | 4181 | -0.432 | -0.4820 | No |
| 42 | MAP1B |  |  | 4298 | -0.472 | -0.4979 | No |
| 43 | NFKB1 |  |  | 4313 | -0.476 | -0.4941 | No |
| 44 | LTBP1 |  |  | 4322 | -0.477 | -0.4892 | No |
| 45 | NOTCH2 |  |  | 4355 | -0.488 | -0.4887 | No |
| 46 | SYNE1 |  |  | 4397 | -0.500 | -0.4898 | No |
| 47 | FHL2 |  |  | 4451 | -0.521 | -0.4929 | No |
| 48 | TJP1 |  |  | 4489 | -0.536 | -0.4928 | No |
| 49 | ADORA2B |  |  | 4535 | -0.556 | -0.4939 | No |
| 50 | NR3C1 |  |  | 4673 | -0.630 | -0.5116 | No |
| 51 | FZD2 |  |  | 4719 | -0.658 | -0.5113 | No |
| 52 | BDNF |  |  | 4795 | -0.707 | -0.5161 | No |
| 53 | NEK7 |  |  | 4807 | -0.719 | -0.5085 | No |
| 54 | PDLIM5 |  |  | 4907 | -0.820 | -0.5164 | No |
| 55 | IGFBP5 |  |  | 4978 | -0.897 | -0.5176 | Yes |
| 56 | MYC |  |  | 5020 | -0.972 | -0.5123 | Yes |
| 57 | KCNMA1 |  |  | 5031 | -0.990 | -0.5008 | Yes |
| 58 | DLC1 |  |  | 5036 | -1.013 | -0.4879 | Yes |
| 59 | LPAR1 |  |  | 5042 | -1.024 | -0.4750 | Yes |
| 60 | CITED2 |  |  | 5045 | -1.026 | -0.4615 | Yes |
| 61 | ANXA2 |  |  | 5055 | -1.036 | -0.4492 | Yes |
| 62 | MET |  |  | 5073 | -1.080 | -0.4378 | Yes |
| 63 | ANXA4 |  |  | 5106 | -1.172 | -0.4281 | Yes |
| 64 | COL11A1 |  |  | 5110 | -1.178 | -0.4127 | Yes |
| 65 | COL5A2 |  |  | 5112 | -1.189 | -0.3968 | Yes |
| 66 | RND3 |  |  | 5128 | -1.230 | -0.3830 | Yes |
| 67 | DUSP1 |  |  | 5138 | -1.269 | -0.3675 | Yes |
| 68 | ATP2B4 |  |  | 5139 | -1.270 | -0.3503 | Yes |
| 69 | MT1E |  |  | 5154 | -1.323 | -0.3351 | Yes |
| 70 | SERPINE1 |  |  | 5161 | -1.377 | -0.3176 | Yes |
| 71 | EFEMP1 |  |  | 5199 | -1.550 | -0.3037 | Yes |
| 72 | TFPI |  |  | 5202 | -1.557 | -0.2830 | Yes |
| 73 | NRP1 |  |  | 5211 | -1.609 | -0.2627 | Yes |
| 74 | SNAI2 |  |  | 5241 | -1.903 | -0.2425 | Yes |
| 75 | F3 |  |  | 5242 | -1.927 | -0.2164 | Yes |
| 76 | MGLL |  |  | 5249 | -2.003 | -0.1904 | Yes |
| 77 | PDGFRB |  |  | 5253 | -2.045 | -0.1633 | Yes |
| 78 | DAB2 |  |  | 5261 | -2.217 | -0.1346 | Yes |
| 79 | CAV1 |  |  | 5279 | -2.605 | -0.1026 | Yes |
| 80 | COL3A1 |  |  | 5292 | -3.630 | -0.0557 | Yes |
| 81 | COL1A1 |  |  | 5294 | -4.144 | 0.0002 | Yes |
Table: GSEA details [plain text format]

  

Fig 2: HALLMARK\_UV\_RESPONSE\_DN: Random ES distribution      
 Gene set null distribution of ES for **HALLMARK\_UV\_RESPONSE\_DN**
